# Inverting the Facing-the-Viewer Bias for Biological Motion Stimuli

**DOI:** 10.1177/2041669517750171

**Published:** 2018-01-09

**Authors:** Séamas Weech, Nikolaus F. Troje

**Affiliations:** Department of Psychology, Queen's University, Kingston, ON, Canada; Department of Psychology, Queen's University, Kingston, ON, Canada; Department of Biology, Queen's University, Kingston, ON, Canada; School of Computing, Queen's University, Kingston, ON, Canada; Centre for Neuroscience Studies, Queen's University, Kingston, ON, Canada; Séamas Weech is now at the Department of Kinesiology, University of Waterloo, Waterloo, ON, Canada.

**Keywords:** biological motion, facing-the-viewer bias, perceptual ambiguity, inversion effect

## Abstract

Depth-ambiguous point-light walkers are most frequently seen as facing-the-viewer (FTV). It has been argued that the FTV bias depends on recognising the stimulus as a person. Accordingly, reducing the social relevance of biological motion by presenting stimuli upside down has been shown to reduce FTV bias. Here, we replicated the experiment that reported this finding and added stick figure walkers to the task in order to assess the effect of explicit shape information on facing bias for inverted figures. We measured the FTV bias for upright and inverted stick figure walkers and point-light walkers presented in different azimuth orientations. Inversion of the stimuli did not reduce facing direction judgements to chance levels. In fact, we observed a significant facing away bias in the inverted stimulus conditions. In addition, we found no difference in the pattern of data between stick figure and point-light walkers. Although the results are broadly consistent with previous findings, we do not conclude that inverting biological motion simply negates the FTV bias; rather, inversion causes stimuli to be seen facing away from the viewer more often than not. The results support the interpretation that primarily low-level visual processes are responsible for the biases produced by both upright and inverted stimuli.

## Introduction

**Figure 1.**
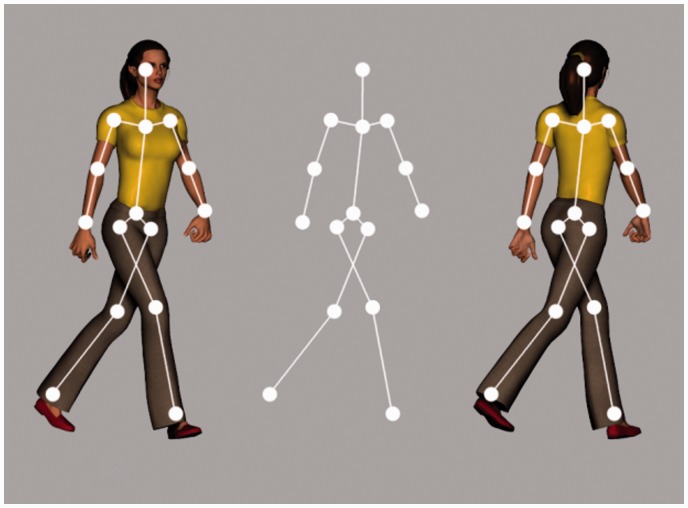
Depth ambiguity in biological motion. Both towards (left) and away (right) views correspond to the stick figure (middle).

Point-light walkers are often used to show the robust ability of the visual system to recover a wealth of information from the structure and kinematics of a human in motion (e.g., Johansson, 1973, 1976; Pollick, Kay, Heim, & Stringer, 2005; Runeson & Frykholm, 1981; Troje, 2008). Point-light depictions of biological motion are depth ambiguous in orthographic projection as they contain no explicit information about the depth ordering of the points that represent parts of the body (see Figure 1). There is a resulting perceptual bistability with respect to the facing direction of a point-light walker projected in the fronto-parallel plane such that the walker can be seen to face towards or away from the viewer (Vanrie, Dekeyser, & Verfaillie, 2004). Despite the fact that either interpretation is perceptually plausible, point-light walkers are subject to a strong bias to be seen as facing towards the viewer (facing-the-viewer [FTV] bias; see for e.g., Schouten, Troje, & Verfaillie, 2011; Vanrie et al., 2004). The first report of the bias proposed a social or biological cause (Vanrie et al., 2004). Figures that appear to be human might be perceived as facing-the-viewer more often due to a desire to prepare oneself for situations that require social interaction or to deal with a threatening situation (e.g., the figure is approaching in order to attack the observer). Support for this theory emerged from a study where FTV bias was reduced after undergoing muscle relaxation and physical exercise (Heenan & Troje, 2014, 2015). The decrease in social anxiety that arose from these techniques was believed to mediate this reduction in FTV bias.

The sociobiological explanation of FTV bias requires that the process of resolving depth order of body features takes place at a later stage than the perception of animacy in the figure. In this case, the two-dimensional (2D) projection of the figure is first matched with a stored internal template. Only then is three-dimensional (3D) form resolved using a sociobiological bias to perceive other humans as facing towards and not away. An alternative shape-driven theory of FTV bias has focused on the influence of low-level stimulus properties at an early stage of visual processing (Weech, McAdam, Kenny, & Troje, 2014). According to this theory, the process of resolving depth ordering takes place in a bottom-up manner: The depth order of point-lights is resolved first of all, leading to a hierarchical integration of the surface shape of body parts, and finally an overall facing direction judgement for the figure that is contingent on the depth ordering of features. The perception of animacy takes place after the resolution of featural depth order. The theory draws upon Marr's object recognition model regarding how 3D information is derived from 2D images (see Marr, 1982; Marr & Nishihara, 1978). In the case of point-light walkers, this model suggests that 2D features such as dots or lines are first identified and are then assigned local surface orientations in order to construct what Marr labelled the 2½ D sketch (Marr, 1982). The final stage is to fit a 3D surface by integrating across local surface orientations. The proponents of this shape-driven theory of FTV bias have reasoned that the bias emerges at the level of the 2½ D sketch, where a preference exists for the visual system to construct surface representations that are convex with respect to the line of sight.

The reasoning behind why such a bias would generate results consistent with FTV bias is outlined in brief here (for more details, see Weech et al., 2014). In the case of a point-light display that represents a human walker facing towards the viewer, the upper body shape is characterised by the curvature of the arms away from the viewer and the lower body shape by the curvature of the legs towards the viewer. Observers appear to adopt a coherent interpretation based mainly on the facing direction that is implied the bottom half of the display: that is, the legs which are convex when the figure faces the viewer. Note that a conflict exists between the direction implied by the legs and the arms if both are assumed to be convex. Given the physically implausibility of a walker that faces in both directions at once, adopting this interpretation as a final percept appears to be highly unlikely.

The low-level explanation of FTV bias outlined earlier was motivated by the observation that FTV bias strongly depends on the representational style of biological motion stimuli (Weech et al., 2014). The authors found that socially relevant, depth-ambiguous human stimuli are not always sufficient to generate FTV bias. In contrast to stick figure walkers, depth-ambiguous silhouettes of a walking human were not seen facing towards or away more often than chance. Since the high-level sociobiological properties of human silhouettes and point-light walkers are similar, these results were taken to signify that FTV bias operates at a low level of representation. Specifically, the bias is likely to emerge at the level of processing where the surface normals of the figure are assigned during construction of the 2½ D sketch. This assignment process differs fundamentally between point-light walkers and stick figure walkers. In stick figures, the orientation of each surface normal is ambiguous with respect to the line of sight, but in silhouettes, the surface normals of the bounding contour are unambiguously defined as lying in the image plane. As such, any bias towards resolving surface normals of features would influence perception of point-light walkers but not silhouettes.

Another observation that challenges the sociobiological explanation for FTV bias is that stimuli with pronounced curvature of the arms and legs were seen facing-the-viewer much more than figures with unbent arms and legs, for which FTV bias was not observed (Weech et al., 2014). Given the lack of ambiguous surface curvature to resolve in such stimuli, the bias for surface convexity did not appear to influence facing direction judgements.

Finally, human silhouettes with dots added to parts of the body where surface curvature was ambiguous resulted in small but reliable biases that depended on the location of the dots. Here, the addition of dots encouraged the inclusion of a shape convexity bias to the process of resolving surface normals. When small dots were positioned on the middle of the legs, the silhouettes were seen more often facing-the-viewer; when positioned on the middle of the arms, the silhouettes appeared more often to face away from the viewer. The results taken together confirmed the idea that there is a strong shape component to FTV bias which is likely to be more related to FTV bias than the social relevance of biological motion stimuli. Given that several static stimuli produced strong FTV bias, the results dispute the importance of motion, biological or otherwise, in the FTV bias. This is in contrast to research that found a diminished FTV bias when local motion cues were absent from point-light displays (de Lussanet & Lappe, 2012).

Another study examined how deconstructed point-light walkers are perceived and also concluded that FTV bias may result from low-level properties of point-light walkers. Schouten et al. (2011) presented observers with top- and bottom-half constituent parts of point-light walkers and found that the bottom half was perceived as facing-the-viewer as often as the full point-light walker, whereas the top half of point-light walkers was most often seen facing away from the viewer. This result supports the idea that surface shape, which differs between the lower and upper halves of the figures, plays a key role in the FTV bias.

One finding that has proven incompatible with the theory of a shape-driven FTV bias is the effect of stimulus inversion on facing direction judgements (Vanrie et al., 2004). The authors presented point-light walkers upside down and found that the strong FTV bias for upright figures disappeared completely upon inversion. This finding has been taken to show that the presence of FTV bias relies upon perceiving a human figure per se, and that disrupting social relevance through inversion diminishes the bias. Given that the shape of the figure remains the same upon inversion, a bias driven mainly by shape could be expected to be equivalent in magnitude between the upright and inverted walkers. However, inversion affects a number of other aspects of biological motion processing, and in particular, the ability to resolve the overall shape of the stimulus. It might be that the effect of inversion upon FTV bias can be attributed to the *shape inversion effect* (Troje & Westhoff, 2006). Body shape is thought to be resolved through a template matching process (Lange, Georg, & Lappe, 2006). Inversion of the stimulus makes this process more difficult, as some kind of transformation to the stimulus or the stored internal model must take place before a match can occur. Disrupting the process of shape estimation could pose a candidate for the deleterious effect of inversion on FTV bias that was reported by Vanrie et al. (2004). If this was the case, the addition of information that would help to resolve shape could lead to a reemergence of FTV bias for inverted figures. A similar stimulus to the point-light display but which includes featural information about shape is the stick figure representation of a walker. This stimulus is subject to a similar degree of FTV bias to the point-light display and also has connecting lines between the dots that help to specify local shape. However, previous experiments have not assessed whether or not this stimulus is subject to FTV bias when inverted.

It could also be the case that inverted stimuli with explicit shape would not be subject to a facing-the-viewer bias but instead would be seen more often facing away from the viewer. This would be expected if observers prioritise the part of the body located in the lower visual field when making assessments of walker facing direction, given that inversion of the stimulus renders the arms in the lower half of the display and the legs in the upper half. The arms are associated with a convex shape when the biological motion stimulus is in the facing away orientation, and as such, using information from the arms to guide judgements would cause the stimulus to appear to face away from the viewer (Schouten et al., 2011). Existing evidence shows that the lower half of biological motion displays is preferentially used to derive information in facing direction tasks and that this preference remains even when stimuli are inverted (Hirai, Chang, Saunders, & Troje, 2011). In addition, it is well documented that observers preferentially obtain information from the lower visual field in a variety of perceptual judgements (Danckert & Goodale, 2003). If FTV bias for upright stimuli is caused primarily by a preference for the lower half of the stimulus, we would expect to observe a facing away bias for inverted stick figures.

We designed an experiment to assess whether stick figures, for which explicit shape is provided, are subject to FTV bias or facing away bias when inverted. We compared the FTV bias for stick figure walkers and point-light walkers in upright and inverted configurations. We expected to observe a facing bias for inverted stick figures, either a FTV bias if the shape inversion effect is responsible for reducing the facing bias in point-light displays or a facing away bias if judgements of facing direction in inverted stick figures are influenced by a lower visual field preference.

## Method

Approval of the study was granted by the Queen's University General Research Ethics Board. We intended to compare our results for point-light displays to those obtained by Vanrie et al. (2004) in order to assess the reliability of results. As such, our experiment was designed to replicate as close as possible the method of Experiment 1 in Vanrie et al. (2004). Any deviations that were taken from the design of Vanrie et al. are identified later in the article.

### Participants

Participants were 40 undergraduate and graduate students at Queen's University, Kingston. Mean age was 19.36 years (*SD* = 1.42). All had normal or corrected to normal vision. Each participant gave informed written consent before the study in accordance with the Declaration of Helsinki.

### Stimuli

We presented walking biological motion figures that represented a bilaterally symmetric average of the gait patterns of 50 men and 50 women based on a Fourier representation as outlined by Troje (2002, 2008). All walkers were rendered both as point-light displays and as stick figures. Half of the stimuli presented were upright and half were inverted. The stimuli were oriented at three different azimuth angles: centre view (0°), three-quarter view to the left (−45°) and three-quarter view to the right (+45°). These projections represented figures that were either facing away or towards the viewer due to the depth ambiguity of the stimuli. In the experiment of Vanrie et al. (2004), stimuli were additionally shown in left and right profile views (−90° and +90°, respectively). We decided to remove these conditions in our study since these projections depicted facing directions that lie in the image plane, whereas we were primarily interested in towards/away facing direction judgements. In addition, the inclusion of the stick figure stimuli doubled the number of trials in our study compared with the original, so we removed these lateral views to reduce the number of trials.

**Figure 2.**
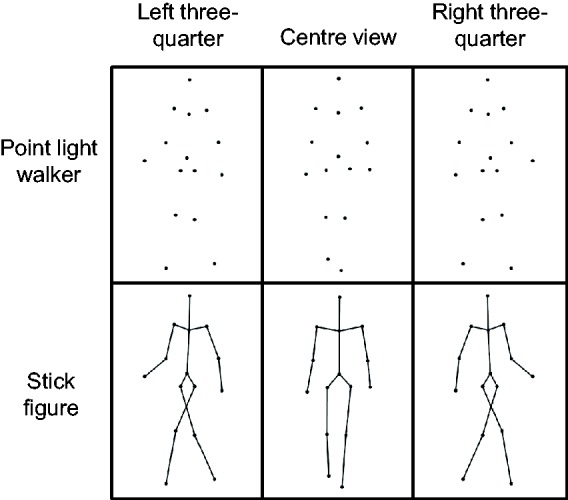
Examples of upright stimuli. Stimuli shown are at azimuth angles of −45/−135, 0/ ±180 and +45/+135, respectively.

The stimuli were presented using Matlab (MathWorks Inc., MA, USA) with the Psychophysics Toolbox (Brainard, 1997) on a 22-in. LCD monitor at a 60 Hz refresh rate. Stimuli subtended visual angles of approximately 7° vertically and 3° horizontally at a viewing distance of 57 cm. All stimuli were white on a black background. Example stimuli are shown in Figure 2.

### Design and Procedure

**Figure 3.**
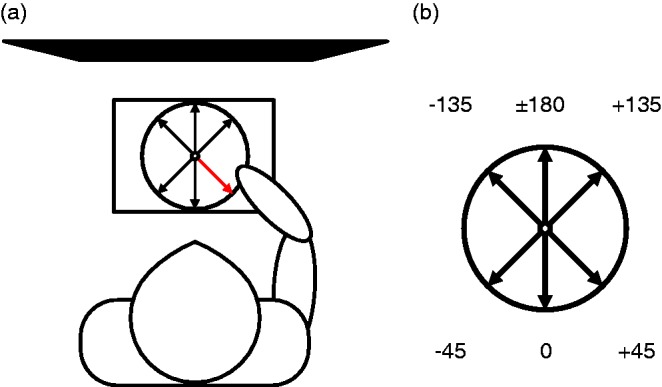
(a) Response apparatus. The red arrow was moveable to one of six directions which were drawn on the circle. (b) Six possible angles of stimulus facing direction.

Adopting the method used by Vanrie et al. (2004), participants were told that they would take part in a task where they must determine the facing direction of point-light and stick figure walkers. Next, they were shown an example of a fronto-parallel projected depiction of upright and inverted point-light displays on paper. The experimenter then described the apparatus with which the participant would make their responses. The apparatus is depicted in Figure 3. This consisted of a circular shape drawn onto cardboard, with lines indicating one of six directions: the centre view, three-quarter views to the left and to the right and the mirror flipped versions of these directions about the image plane. A moveable arrow was attached to the centre of the circle for the purpose of indicating which of the six directions the walker faced in each trial. Participants were instructed not to try to respond with an equal number of facing directions per direction. Participants were asked to fixate on the midpoint of the stimuli. As an example to represent possible facing directions, participants were shown a projection of a car in three-quarter views to the right, both towards (+45°) and away (+135°). Participants were instructed to maintain use of a chinrest throughout the experiment.

Each trial consisted of two phases. First, the biological motion stimulus appeared and walked for 4 seconds. Second, there was a blank screen for 4 seconds during which the participant made a response by rotating the arrow on the response apparatus to denote the facing direction. The experiment progressed automatically without breaks, with the experimenter recording the responses of the participants in every trial.

The experiment conformed to a 3 × 2 × 2 within-subject design, where the factors were: azimuth (centre view and three-quarter views to the left and right), inversion (upright and inverted) and rendering (point-light displays and stick figures). The inversion and rendering factors were blocked. The azimuth changed randomly from trial to trial. The order of the four blocks was counterbalanced across participants using a Latin Square design. Each stimulus was repeated 20 times resulting in a total of 240 trials. The total duration was approximately 45 minutes including instruction and debriefing.

We computed a measure of response accuracy from the proportion of correct identifications of the azimuth angles of walkers, regardless of whether it was perceived as facing towards or away. For example, a correct response for a left three-quarter view walker would be both the −45 or −135 arrows on Figure 3(b). These responses would indicate facing towards or facing away, respectively.

We measured FTV bias using the proportion of trials where the walkers were reported to be facing towards the viewer (−45, 0, or +45 directions on Figure 3(b)), as opposed to facing away (−135, ±180, or +135 directions on Figure 3(b)). We call this the proportion FTV. If all stimuli were seen as FTV, the proportion FTV would be 1, and if all were seen facing away, the proportion FTV would be 0. Chance response levels would result in a proportion FTV of 0.5.

## Results

### Response Accuracy

Observers were highly accurate in all conditions. Accuracy was similar for both point-light walkers (upright: *M* = 99, *SEM* = .003 and inverted: *M* = 97, SEM = .008) and stick figures (upright: *M* = 97, *SEM* = .006 and inverted: *M* = 97, *SEM* = .009). No interactions or main effects were observed for accuracy.

### FTV Bias

We conducted a three-way analysis of variance (ANOVA) on the proportion FTV for the factors of azimuth, inversion and rendering. There was no significant three-way interaction (*p* = .46) and no significant two-way interactions (*p*s ≥ .07).

#### Effect of rendering and inversion

**Figure 4.**
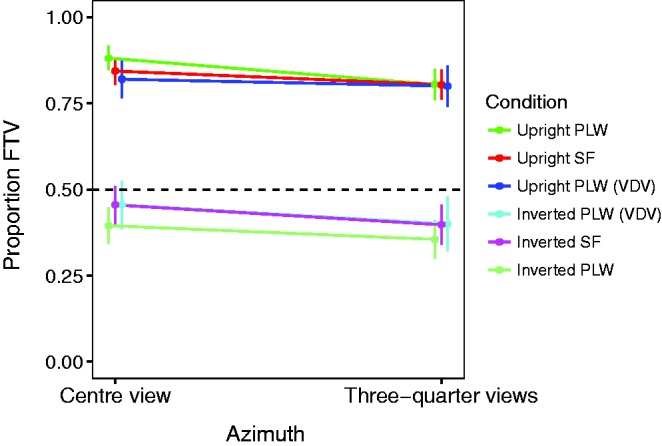
Proportion of FTV responses for upright and inverted PLW and SF. VDV = Data replotted from Vanrie et al. (2004). Dotted line indicates point of subjective equivalence between facing towards and away from the viewer. Error bars are standard errors of the means. PLW = point-light walkers; SF = stick figures.

Proportion FTV responses are plotted in Figure 4. There was a clear main effect of inversion, *F*(1, 39) = 43.17, *p* < .001, $\eta_{\text{p}}^{2}=.53$. Mean proportion FTV for upright figures was 0.82 and for inverted figures was 0.39. One sample two-tailed *t* tests compared with the 0.5 level (point of subjective equivalence, PSE) showed the FTV bias was significant for upright figures, *t*(39) = 15.44, *p* < .001, and the facing away bias was significant for inverted figures, *t*(39) = 2.91, *p* = .005. There was no main effect of rendering, *F*(1, 39) = 0.91, *p* = .35, $\eta_{\text{p}}^{2}=.02$

Upright stimuli were seen FTV at a proportion of 0.77 or higher in all conditions, while the inverted stimuli were seen FTV at a proportion of 0.43 or lower in all conditions. We examined whether facing bias was significant for each condition separately using two-tailed one-sample *t* tests compared with the PSE of 0.5 (see Table 1). All upright stimulus conditions showed significant FTV biases, and four of the six inverted stimulus conditions produced significant facing away biases. The two conditions that did not show significant bias were the inverted stick figures in the central view and the right three-quarter view.

**Table 1.** Mean Proportion FTV and One-Sample *t* Tests Against 0.5 for All Conditions.

Inversion	Azimuth	Rendering	Proportion FTV	*SEM*	*t*(39)	*p*
Upright	Left	Stick figure	0.770	0.046	5.91	< .001***
		Point-light	0.776	0.050	5.56	< .001***
	Centre	Stick figure	0.869	0.034	10.88	< .001***
		Point-light	0.906	0.028	14.77	< .001***
	Right	Stick figure	0.889	0.031	12.42	< .001***
		Point-light	0.884	0.030	12.79	< .001***
Inverted	Left	Stick figure	0.351	0.054	2.75	.009**
		Point-light	0.311	0.053	3.56	.001**
	Centre	Stick figure	0.432	0.054	1.25	.220
		Point-light	0.370	0.052	2.49	.017*
	Right	Stick figure	0.394	0.059	1.79	.081
		Point-light	0.349	0.055	2.75	.009**

*p < .05; **p < .01, ***p < .001.

**Figure 5.**
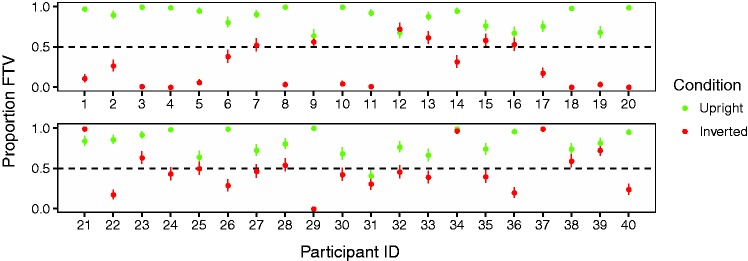
Individual proportion FTV for upright and inverted stimuli. Dotted line indicates point of subjective equivalence between towards and away. Error bars are standard error of the means.

Inspection of individual participant data revealed that many participants consistently observed the upright stimuli as FTV and the inverted stimuli as facing away. Individual data are plotted in Figure 5.

#### Effect of azimuth

**Figure 6.**
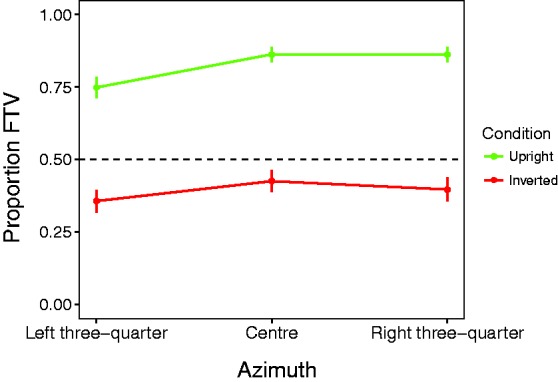
Proportion FTV for each level of the azimuth factor. Dotted line indicates point of subjective equivalence between towards and away. Error bars are standard error of the means. FTV = facing-the-viewer.

Responses across the three levels of azimuth did not meet the assumption of sphericity—Mauchly's *W* = 0.66, approximate *Χ*^2^(2) = 15.82, *p* < .001—so a Greenhouse-Geisser correction was applied to the repeated measures ANOVA on azimuth. The test revealed a main effect of azimuth, *F*(1.5, 58.2) = 4.49, *p* = .024, $\eta_{\text{p}}^{2}=.10$. A follow-up analysis with paired *t* tests indicated that the left three-quarter view stimuli were seen as FTV less often than those in the centre view condition, *t*(39) = 3.30, *p* = .002. The right three-quarter view condition was not different from either the centre view condition, *t*(39) = 0.57, *p* = .57, or the left three-quarter view condition, *t*(39) = 1.85, *p* = .07. Data for the azimuth conditions are plotted in Figure 6.

## Discussion

Here, we have found that both stick figure walkers and point-light walkers produce a significant facing away bias when inverted. We initially predicted that adding explicit shape information using connecting lines between points would produce FTV bias if observers tended to use the legs to infer facing direction or a facing away bias if the lower half of the display was dominant. The fact that we observed facing away bias for almost all inverted stimuli provides strong evidence that facing bias is primarily caused by a convexity bias operating at a low level of visual processing. The results complement several studies that highlight the crucial role of low-level stimulus features in the FTV bias (de Lussanet & Lappe, 2012; Schouten et al., 2011; Weech et al., 2014). We also documented for the first time that there is a small but significant dependency of FTV bias on the viewpoint of the biological motion walkers.

### Inversion of Biological Motion Stimuli Produces a Facing Away Bias

For upright stimulus conditions, the data we obtained were broadly similar to the results of Vanrie et al. (2004), which is not unexpected given that we closely replicated their methodology. However, our data indicated a significant facing away bias in inverted biological motion stimuli which has not been previously identified. This bias was observed not only for the stick figure walkers we presented in this experiment but also for point-light walkers that were similar to those used in several other studies. While Vanrie et al. (2004) did not report a significant bias for inverted point-light walkers, this might have been due to a difference in statistical power. In replicating the previous study, we included twice as many participants and also doubled the number of trial repetitions per condition.

The fact that we found a significant facing away bias for the inverted stimuli supports the low-level explanation of FTV bias for biological motion. Participants appear to have been preferentially guided in facing judgements by the shape of the part of the walker that was located in the lower part of the display in both the upright and inverted cases. This preference would result in FTV bias for the upright stimuli because a convexity bias for the legs causes the body to face the viewer. As well, the tendency for the lower visual field to guide judgements would produce a facing away bias for inverted stimuli because a convex shape bias for the arms would encourage the observer to adopt the interpretation that the stimulus faces away from the viewer.

Previous research has shown that the lower part of a point-light walker is preferentially used to guide judgements of facing orientation in depth for upright figures (Schouten et al., 2011). Importantly, this preference does not switch to the upper visual field when point-light walkers are inverted (Hirai et al., 2011). Given that the physics of walking on a ground plane are dictated by gravity, the lower part of the visual field is typically the most useful area for gathering information about facing direction. In other aspects of visual perception, there is clear evidence for the existence of a visual filter that causes the lower visual field to take preference (see Danckert & Goodale, 2003, for a review). Motion, contrast, figure-ground, and contour shape are processed quicker and more accurately in the lower visual field when compared to the upper field (Levine & McAnany, 2005; Previc, 1990; Rubin, Nakayama, & Shapley, 1996; Schmidtmann, Logan, Kennedy, Gordon, & Loffler, 2015). Some authors have suggested that the lower visual field preference is due to increased attentional resolution for this part of the visual field, especially when action execution is involved in the task (He, Cavanagh, & Intriligator, 1996; Kraft et al., 2011; Rossit, McAdam, Mclean, Goodale, & Culham, 2013; Schmidtmann et al., 2015). In this context, it may be the case that preferential attention towards the lower half of biological motion stimuli – both upright and inverted – produced the pattern of results we observed here.

The strength of the FTV bias for upright figures was about 20% stronger than the facing away bias for inverted figures, meaning that inverting the figure does not completely invert the bias. This asymmetry is likely to reflect the configural inversion effect that has been identified several times in biological motion research (Bertenthal & Pinto, 1994; Chang & Troje, 2009; Sumi, 1984). The difference in the magnitude of bias for upright and inverted stimuli could also reflect the contribution of social relevance to the strength of the FTV bias for upright figures, as was proposed by Vanrie et al. (2004) and later supported by Heenan and Troje (2014, 2015).

### Facing Bias Depends on the Stimulus Azimuth

Here, we documented a second novel finding: Facing direction judgements depend on the azimuth orientation of stimuli. If a stimulus was presented in the left three-quarter view, it was seen less often facing towards the viewer than in the centre view. Although the left-facing condition produced a smaller proportion of FTV responses than the right-facing condition, this difference did not reach statistical significance (*p* = .07). It is important to note that the left- and right-view conditions were visually identical apart from a mirror flip about the medial plane.

It is unclear why a left-right asymmetry might exist for the FTV bias. The result cannot be explained by differences in response accuracy, given that there were no significant differences in response accuracy between the azimuth conditions. We also examined if handedness could be the cause of the result, given that most of our participants were right handed and because a link between handedness and task performance for stimuli presented in different visual hemifields has been previously identified (Marzoli, Prete, & Tommasi, 2014). Analysis of data from the four left-handed participants revealed a similar pattern of results to those obtained from the right-handed observers. It is possible that further exploration of this unexpected finding may draw inspiration from research on cerebral laterality, long known to exist in face perception (Broman, 1978; Ellis & Shepherd, 1975; Hilliard, 1973) which shares a right-hemispheric preference with biological motion perception. The phenomenon of *pseudoneglect*, where normal individuals display preferential attention for the left side of the visual hemifield (Bellgrove, Dockree, Aimola, & Robertson, 2004; Bowers & Heilman, 1980; Manly, Dobler, Dodds, & George, 2005; Nicholls, Bradshaw, & Mattingley, 1999), may also help to explain the finding. However, to our knowledge, no such finding has previously been observed in biological motion perception.

## Conclusion

A growing body of literature has highlighted the multiplicity of factors underlying the phenomenon of FTV bias. The results of the current study conform to the existence of a shape-driven bias, in line with other experiments which proposed similar low-level mechanisms at play (de Lussanet & Lappe, 2012; Schouten et al., 2011; Weech et al., 2014). The lower half of point-light and stick figure walkers appeared to carry most importance for observers who were tasked with resolving the facing direction of figures, even when the figures were inverted. These results support previous research on the significance of the lower half of point-light displays for judging facing direction (Hirai et al., 2011; Schouten et al., 2011). Preliminary evidence for a perceptual anisotropy in facing direction judgements was also identified, although further examinations should be carried out to identify an explanation for this effect. The findings show once more that inversion effects such as the one observed here can help to reveal the characteristics of the visual filters that support perception.
